# Challenges in Anaesthetic Management in a Case of Facial Plexiform Neurofibromatosis Posted for Debulking Surgery

**DOI:** 10.7759/cureus.34406

**Published:** 2023-01-30

**Authors:** Sambit Dash, Neeta Verma, Sheetal Madavi, Jui A Jadhav, Aruna Chandak

**Affiliations:** 1 Department of Anesthesiology, Jawaharlal Nehru Medical College, Acharya Vinoba Bhave Rural Hospital, Datta Meghe Institute of Medical Sciences (Deemed University), Wardha, IND

**Keywords:** neurofibromatosis ii, anesthesiology, difficult airway management, plastic and reconstructive surgery, plexiform neurofibromatosis

## Abstract

A patient diagnosed with facial plexiform neurofibromatosis type 1 who is 18 years old and is scheduled for tumour resection and debulking surgery of his face is the subject of this study. The purpose of this paper is to describe the anaesthetic treatment that was administered to this patient. In addition, we analyze the relevant literature, with a particular emphasis on the implications of modifying neurofibromatosis to achieve anaesthesia. Our patient was found to have multiple huge tumours all over his face. When he first arrived, he experienced cervical instability as a result of the enormous mass on the back of his head and in the region of his scalp. He also expected to have difficulty maintaining an airway and breathing through a bag and mask. To protect the patient's airway, a video laryngoscopy was performed, and a difficult airway cart was maintained on standby in the event it was required. In conclusion, the purpose of this case study was to demonstrate the relevance of comprehending the one-of-a-kind anaesthetic requirements of persons diagnosed with neurofibromatosis type 1 who are about to undergo surgical procedures.

Neurofibromatosis is an extremely uncommon kind of disease that requires the anesthesiologist's undivided attention in surgical settings. Careful pre-operative planning and competent intra-operative treatment are required when dealing with patients who are expected to have difficult airway management.

## Introduction

Neurofibromatosis, often known as NF, is a genetic disease that can lead to the development of both ectodermal and mesodermal tumours. It is a disease that is passed down as an autosomal dominant trait. On the basis of the clinical signs, it may be divided into two different categories: NF1 and NF2 [1]. In the year 1882, Frederich von Recklinghausen published his description of NF1, which led to the illness being referred to as von Recklinghausen's sickness [2]. It has nothing to do with a person's gender or race, and every year it affects somewhere between one in 2000 to 3000 people globally. The mutation on chromosome 17 activates the gene NF-1, which results in the production of a lower quantity of the defective neurofibromin protein. Neurofibromin has a role in the control of cell division as well as the suppression of tumours [3]. Due to the fact that the mutation is completely penetrant, it is possible that each and every patient who has this genotype may, at some point in their lives, exhibit some phenotypic expression; however, the nature of this expression will vary and be unpredictable. Hereditary factors are responsible for around half of all instances, while chance mutations are to blame for the other occurrences.

NF1 is the most common form of phakomatosis (neuro-oculo-cutaneous syndrome), and persons with this condition may experience thoracic symptoms anywhere from 10-20% of the time. Upper airway dysfunction can occur when there are intraluminal or extraluminal lesions present, either of which can promote compression of the tracheobronchial tree [4]. To correctly diagnose NF1, seven defining criteria have been uncovered: 1. at least six café-au-lait spots measuring more than 5 mm in children or more than 15 mm in adolescents; 2. at least two neurofibromas of different types or a plexiform neurofibroma; 3. axillary or inguinal freckles; 4. optical gliomas; 5. two or more Lisch nodules; 6. distinct bone lesions; and 7. a first-degree relative who has NF1. It is necessary for at least two of these traits to be present to establish a diagnosis [5]. The incidence of NF2 is one in 33000 to 40000 instances, which is significantly lower than the incidence of NF1. It is a genetic condition that is passed down in an autosomal dominant manner and is caused by changes on chromosome 22. NF2, like NF1, may impact both the ectodermal and the mesodermal tissues of the body, as well as any and all of the body's systems. The diagnostic gold standard is an MRI scan that examines both the brain and the whole spinal canal. Although hearing loss affects the majority of patients, malignancies of the eighth cranial nerve are the most significant diagnosis.

Some children present with symptoms that are similar to those of polio, including atrophy of the muscles in the lower limbs. Approximately 3-5% of individuals will develop a more extensive form of polyneuropathy. The skin characteristics of NF2 patients are noticeably less pronounced than those of NF1 patients. Neurological development and learning are not disrupted in individuals with NF2 in the same way that they are in those with NF1. Due to the fact that the surgical approach to the patient is planned during this stage, the pre-anesthetic assessment of NF patients is an extremely important step [6]. As a consequence of this, it is essential for the anesthesiologist to pay attention to the typical signs and symptoms of NF, as well as its manifestations in the airways and neuroaxis, in order to prepare the anaesthetic approach to satisfy the high performance and safety needs of each individual patient. This condition has important repercussions for the treatment of anaesthesia due to the fact that, depending on the location of the tumours, the patient may present with a difficult airway (AW) for both breathing and intubation. This can be a challenge for the anesthesiologist. If the patient has neurofibromas on the tongue or larynx, as well as masses in the cervical or cranial regions, it may be challenging to place them in the optimal position for the AW approach [7]. Another concern is that it will be linked with other neoplasms, such as pheochromocytomas and carcinoid tumours, which would make the anesthetic-surgical approach more difficult and time-consuming [8,9].

## Case presentation

**Figure 1:**
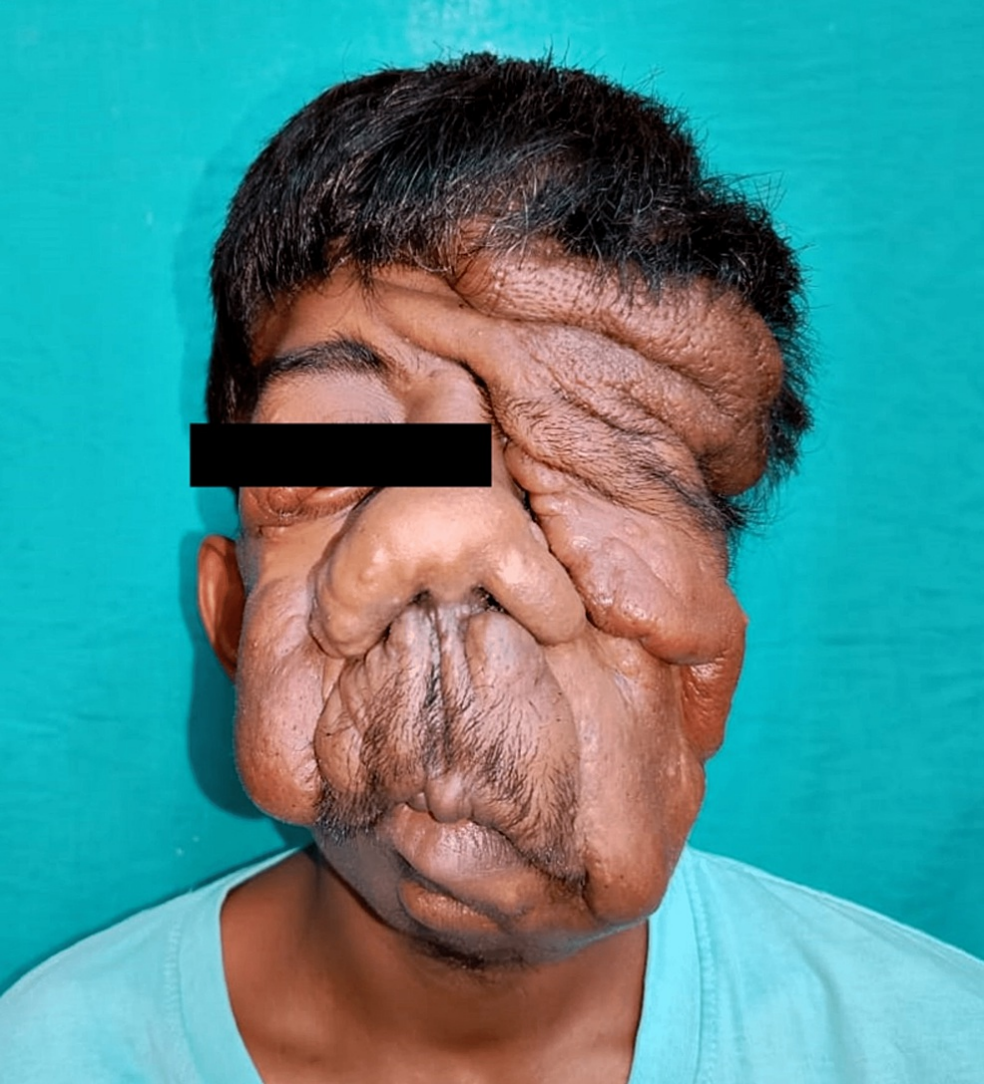
Face of patient clearly showing the excessive growth of tumor, thereby occluding the vision in right eye and causing breathing discomfort to patient due to abnormal growth on nose.

An 18-year-old male patient weighing 45 kg, a diagnosed case of plexiform neurofibromatosis, presented to casualty with a complaint of diminished vision and difficulty in breathing due to excessive tumour growth on the face. The patient had a history of debulking surgery performed on the right upper eyelid and left cheek region seven years back under general anesthesia. On examination patient’s vitals were heart rate (HR) 84 beats per minute, blood pressure (BP) 110/70 mmHg, peripheral oxygen saturation (Spo2) 99% on room air, and respiratory rate (RR) 20 breaths per minute. The patient's physical examination revealed a massive soft tissue swelling involving the right mandibular region and cheek, extending up to the nasolabial fold, pulling down the right angle of the mouth as well as the right eye and the left eye, and partially obstructing the field of vision in both eyes (Figure 1). In addition to axillary freckles and coffee-and-cream macules all over his back, he had a few neurofibromas all over his trunk. A possible cerebral extension was ruled out by magnetic resonance imaging (MRI) of the patient's face.

**Figure 2:**
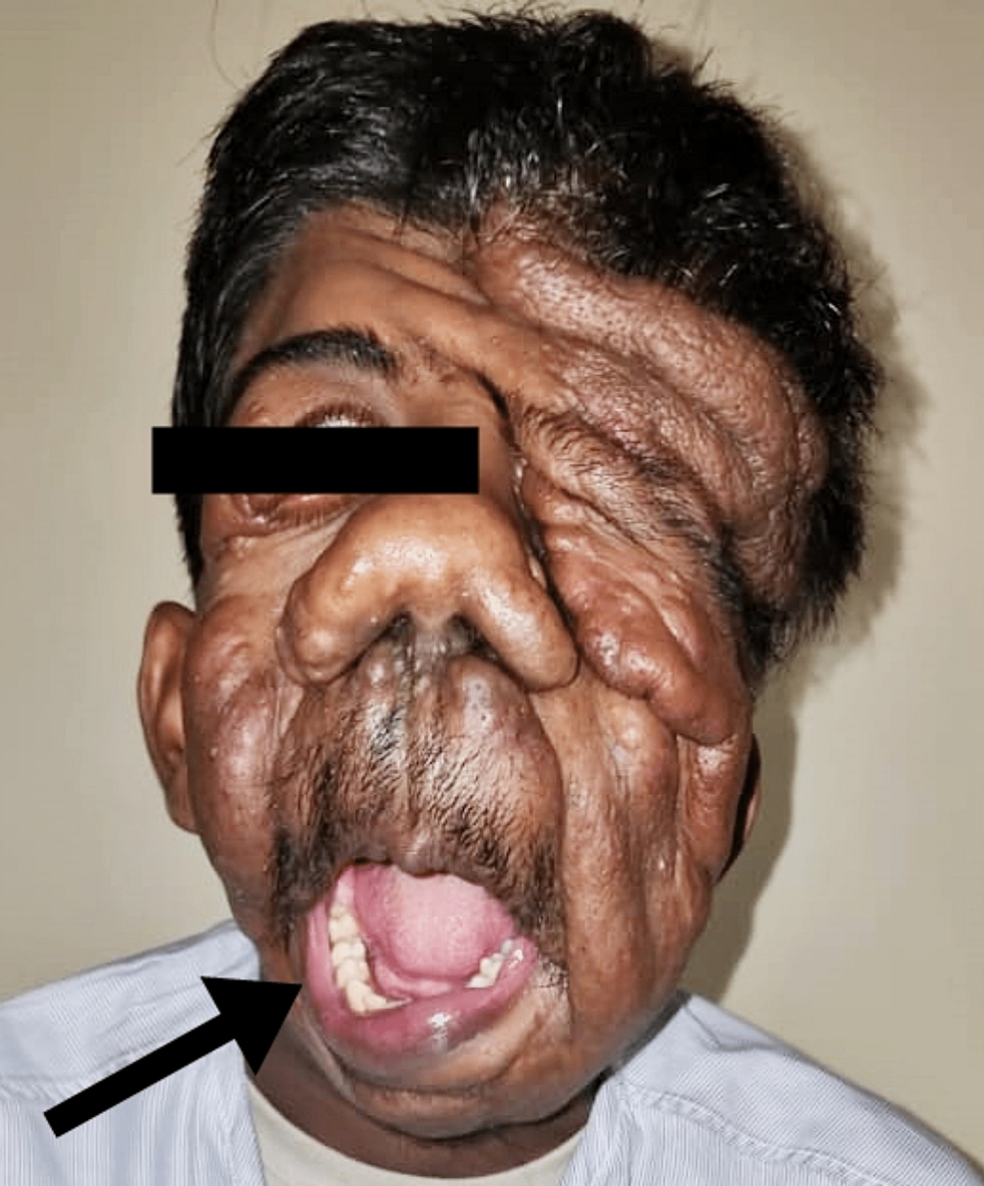
Image showing the restricted mouth opening of the patient indicated by arrows.

**Figure 3:**
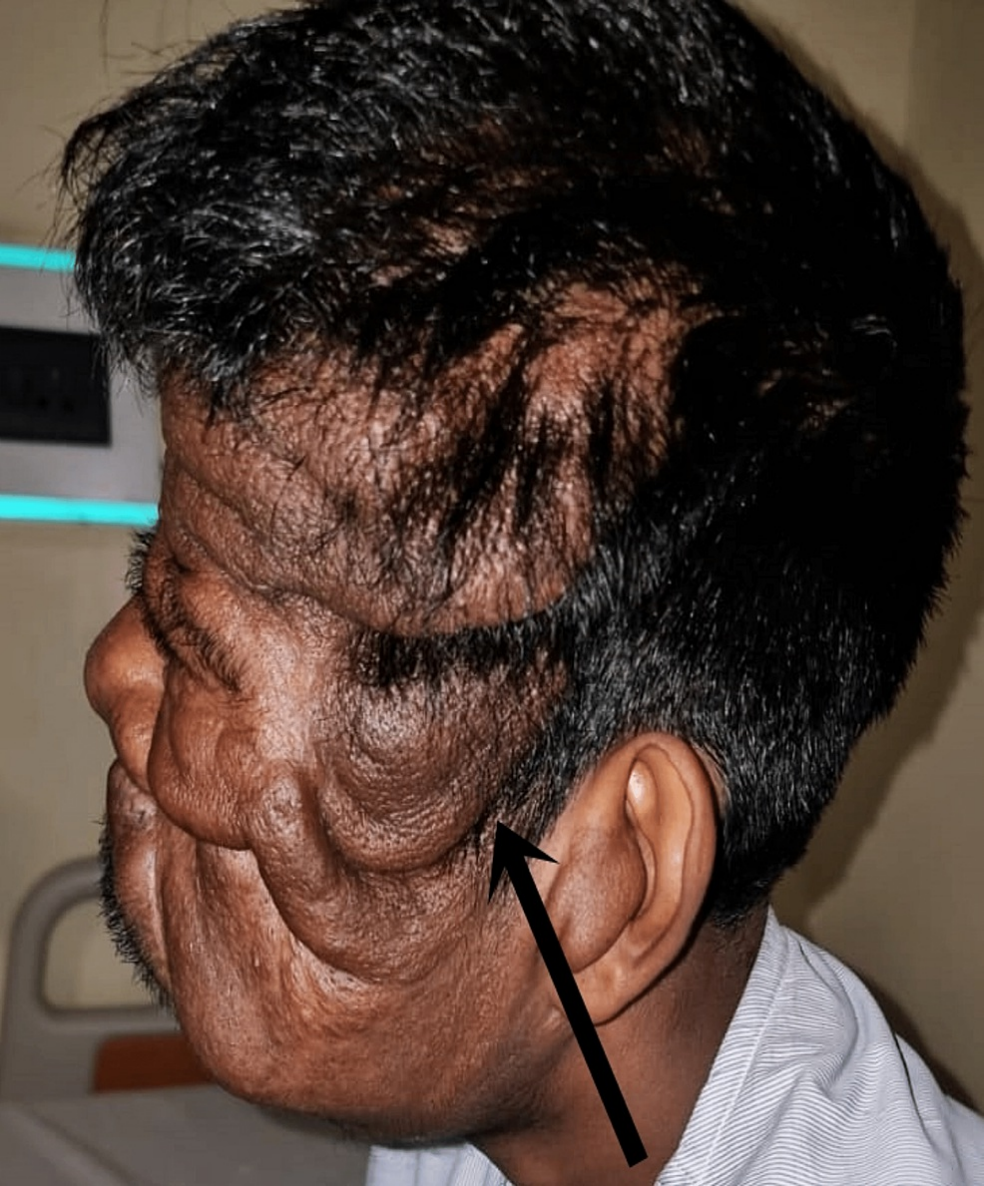
Image showing the generalised growth over cheek, forehead and scalp region indicated by arrows.

The patient was then referred to the plastic surgery department for further management. After assessing the patient they posted the patient for debulking surgery and the patient was sent for a pre-anaesthetic checkup (PAC). During PAC, the patient was categorized under the American Society of Anesthesiologists (ASA) classification grade II. Haemodynamics were recorded to be normal. He denied having allergies and the use of any continuous medications. There was no family history of the disease. Mouth opening (MO) was found to be more than two fingers but less than three fingers with Mallampatti class IV (only hard palate was visible) (Figure 2). The patient had developed small growth of tumours all over the hard palate, making securing of the supraglottic airway insertion difficult, if and when needed during induction of anaesthesia. Difficult bag and mask ventilation was anticipated due to facial abnormalities owing to excessive tumour growth. Tempero mandibular joint (TMJ) movement was found to be adequate but neck extension was restricted by tumour growth on scalp and cervical region (Figure 3). The patient complained of difficulty in breathing and a bedside pulmonary function test (PFT) resulted in a single breath count of 18 and breath holding time of 22 seconds. Bilateral air entry was auscultated to be equal and no added sounds. The patient was categorized into class II as per New York Heart Association (NYHA) classification. There were no alterations in preoperative laboratory tests.

Two units of fresh frozen plasma (FFP), a postoperative intensive care unit (ICU) bed, high-risk consent, arrangement for flexible fibreoptic bronchoscope and video laryngoscopy was advised and fitness was given for surgery. Enhanced recovery after surgery (ERAS) protocol was followed for the patient. Proper preoperative counselling was done, and adequate preoperative nutrition was maintained. The patient was kept nil by mouth for six hours pre-op, with adequate maintenance of IV fluids. After confirming high-risk consent from parents, the patient was shifted onto the operating table. Two 18G peripheral intravenous catheters were secured on either hand. Multiparameter monitors were attached. A fibreoptic flexible bronchoscope was arranged on standby if needed. Video laryngoscopy was planned with a short-acting depolarising muscle relaxant. The patient was premedicated with injection (Inj) pantoprazole 40 mg, Inj glycopyrrolate 0.2 mg, Inj midazolam 1 mg, Inj Fentanyl 50 mcg and induction was done with Inj propofol 100 mg and Inj scoline 100 mg.

The airway was secured with an 8.0 mm cuffed flexo metallic endotracheal tube guided by a video laryngoscope using a bougie to railroad the endotracheal tube. Cormack Lehane (CL) grade was noted to be grade 2b, only the aretynoids and very posterior origin of cords were visible. Once patient took spontaneous respiration post succinylcholine administration, Inj vecuronium 6 mg was administered. The patient was maintained on oxygen, nitrous oxide (2 litres each) and sevoflurane, titrated to maintain a minimum alveolar concentration (MAC) of 0.8 to 1.0. Top-up doses of Inj vecuronium at 1 mg were administered intermittently. Tidal volume (TV) was kept at around 8ml/kg at 350 ml.

**Figure 4:**
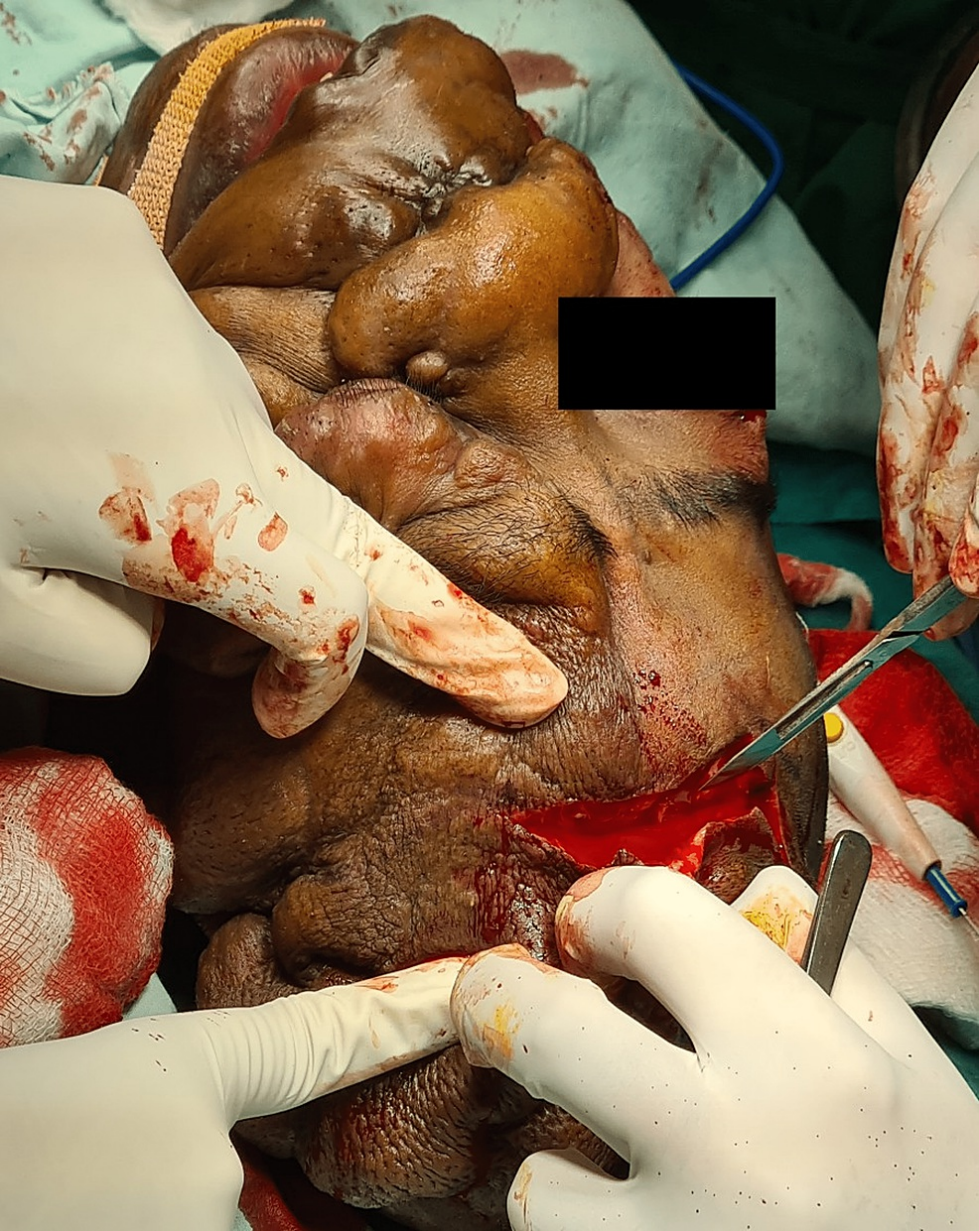
Image showing remarkably high blood loss, particularly during debulking of scalp region.

**Figure 5:**
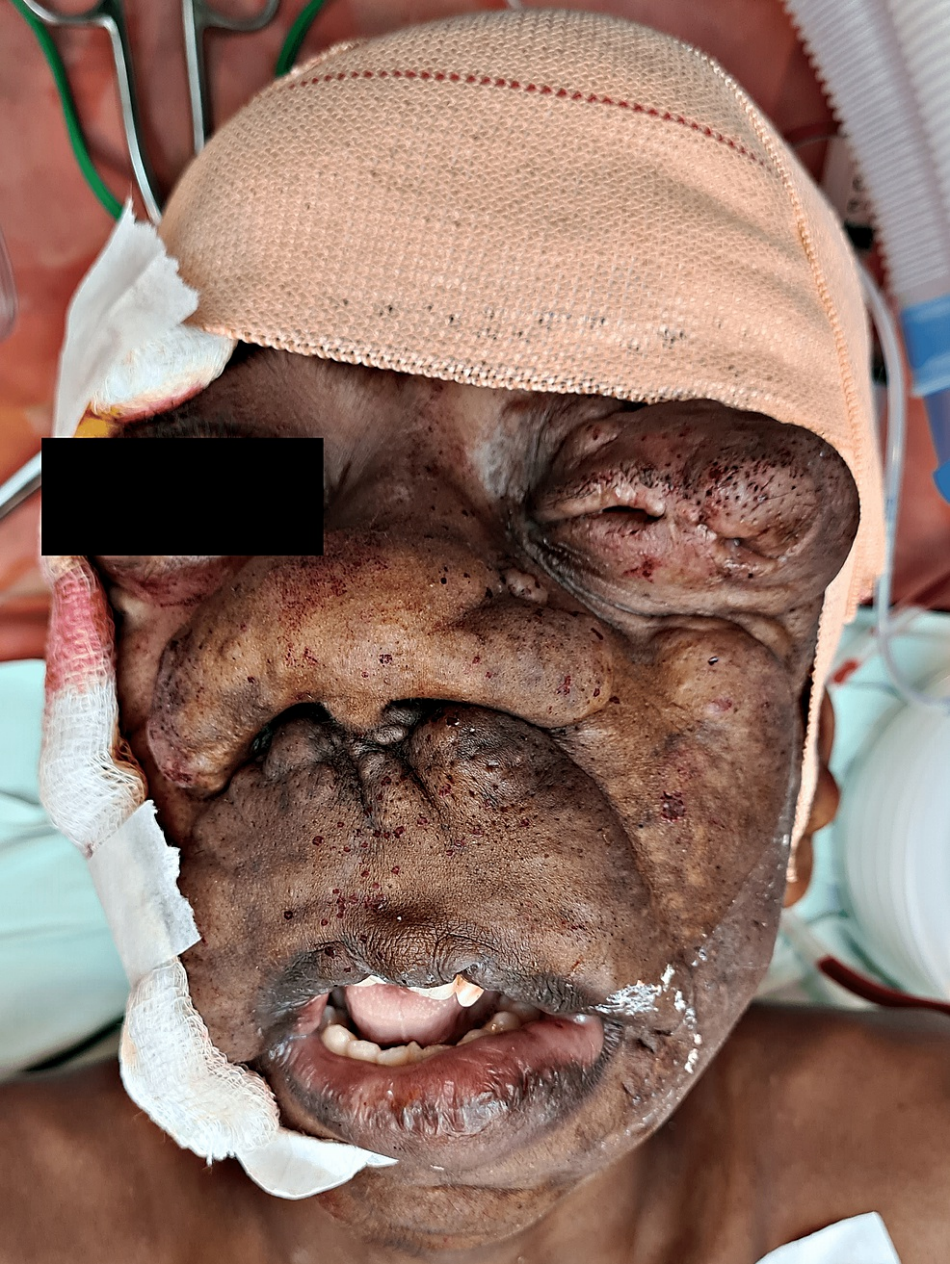
Image of patient post reversal of anesthesia and successful extubation.

Intraoperative blood loss was well anticipated. The allowable blood loss was 800 ml for our patient based on our calculation. Once debulking of the cranial region was started, blood loss was remarkably high (Figure 4). The patient started to show signs of tachycardia and hypotension. Two units of packed red blood cells (PRBC) were issued as soon as possible and transfusion was started. The total blood loss was calculated to be approximately 1400 ml. Two units of PRBCs were transfused over a period of three hours. Haemodynamics were maintained throughout. Tachycardia settled and blood pressure also improved. After the completion of surgery, extubation was not rushed. After the patient was out of muscle relaxant effect, reversal agent Inj neostigmine with glycopyrolate was given at a dose of 0.05 mg/kg of body weight. Patient was allowed to have spontaneous and regular breaths and only after the patient was completely awake, obeying commands and moving all four limbs, the endotracheal tube was deflated and patient was extubated after thorough oral suctioning. Post extubation, the patient was shifted to the surgical intensive care unit (ICU) for further management (Figure 5). Postoperative day 3 (POD 3) patient was discharged from the hospital.

## Discussion

The skin symptoms of NF1 are particularly common in children and adolescents. Café-au-lait spots, axillary and inguinal freckles, and Lisch nodules have unknown clinical relevance. Neurofibromas, on the other hand, are present in 30-50% of cases, grow larger during adolescence and pregnancy, and have a significant risk of malignant transformation, which occurs in only 2-5% of cases but is the major cause of mortality [2]. Although there is some debate on how neurofibromas should be categorised, the cutaneous, subcutaneous, and plexiform types are by far the most frequent. Plexiform neurofibromas have been linked to several unpleasant side effects, including compression of the spinal cord and nerve roots, as well as erosion of the vertebrae. Malignancy can develop inside of a benign nodule, and when it does, the nodule will display some of the features associated with malignancy, such as the expansion or compression of neighbouring tissues. Sarcomas are the kind of tumours that are 100 times more common in this population [10]. Because of the wide variety of comorbidities that can impact several different organs and systems, NF can be a difficult condition for anesthesiologists to manage. Short stature, abnormalities of the bones, and cardiovascular anomalies such as congenital heart malformations, vasculopathy, and hypertension are some of the conditions that are of particular importance for the treatment of anaesthesia and surgery-related aberrations. Cognitive impairments and attention and hyperactivity problems are two conditions that can exist together [2]. Patients who have rhabdomyosarcomas, gastrointestinal stromal tumours (GIST), pheochromocytomas, carcinoid tumours, or ganglioneuromas are more likely to have neurofibromatosis type 1, an inherited genetic disorder. Because diagnosing NF1 is mostly a clinical endeavour, it is essential to do a thorough search for the disease's symptoms before receiving anaesthesia. In addition, to reduce the risk of surgical complications, patients with pigmented patches need to be differentiated from patients with McCune-Albright disease, Peutz-Jeghers disease, and Addison's disease [5]. There are many different consequences of the anaesthetic. About 5% of those diagnosed with NF1 have oral symptoms, which might make normal orotracheal intubation more difficult or perhaps impossible to perform. In addition, a tracheostomy may be required if these warning indications are not recognised before the introduction of anaesthesia. Facial defects can lead to facial asymmetry, which can contribute to issues with breathing and intubation [11]. This is because the bone is present in the face.

Large cervical tumours have the potential to cause cervical instability and airway obstruction due to their interaction with the spinal cord. These tumours make it difficult to treat the airways, which is why awake bronchoscopy intubation is required. This method is regarded as the gold standard approach by the American Society of Anesthesiology when it comes to several different scenarios [12]. It is permissible to take this method if there are signs of a difficult airway or a history of intubation difficulties in the patient. Awake intubation is required when the airways are compromised for any number of reasons, including infection, oedema, hematoma, limited mouth opening, macroglossia, obesity, inability to extend the neck or cervical instability, sleep apnea, Mallampati classification III or IV, protruding or weak teeth, and so on. Inadequate glottic exposure is responsible for the vast majority of the challenges that arise during difficult tracheal intubations. There is a wide range of possibilities when it comes to the incidence rates of difficult direct laryngoscopy and difficult intubation [13]. Although laryngeal exposure by itself does not indicate difficult tracheal intubation [14], the Cormack/Lehane III and Cormack/Lehane IV laryngeal views are often thought to signify difficult tracheal intubation. In these kinds of circumstances, it is possible to gain a Cormack-Lehane I or II view, even though orotracheal intubation might not be effective. Although fiberoptic laryngoscopy (FOL) is still considered the gold standard for tracheal intubation in difficult airways [15,16], the Glidescope (Verthon, Bothell, WA, USA) offers several major benefits. On the other hand, the fiberoptic bronchoscope (FOB) calls for substantial training and isn't always easily accessible. In addition, the Glidescope is designed for the same applications as a conventional laryngoscope and has a shape that is analogous to that instrument. The application of this device is limited in some ways, much like the applications of other devices. Even while the Glidescope is simple to operate, it can be tricky to manoeuvre in some situations, particularly when there is a limited amount of space available. We assume that this is because its angulation is more pronounced than that of a Macintosh laryngoscope. Therefore, in situations when the laryngoscope needs to be positioned in a plane that is more tangent to the tongue (for example, when the mouth opening is particularly tiny or asymmetrical), a blade that has less pronounced curvature may be more useful. Resistance to the advancement of the tracheal tube is an additional limitation. This is something that can be dealt with if the directions provided by the manufacturer are followed. The endotracheal tube is moved forward with the right hand while the stylet is retracted using the left hand. It is recommended that the style be retracted about 2 inches (5 cm). This makes it possible for the tip of the endotracheal tube to enter the larynx, while the stylet ensures that the body of the endotracheal tube remains rigid.

It is possible to inhibit airway reflexes by employing a topical anaesthetic with a lidocaine atomizer, instillation with the use of a bronchoscope, or even the transtracheal route [17]. To induce drowsiness in the patient, the anesthesiologist may choose to utilise the medicine of their choice; nevertheless, it is imperative that the patient's ability to breathe on their own be maintained. The research points to dexmedetomidine as a possible treatment [18]. Because of their inherent physical connections to the spinal cord and spinal canal, the vast majority of patients who have cervical tumours do not experience any symptoms. Despite this, a large number of people report suffering pain, particularly while rotating their bodies. Alterations in neurological function can also be the result of injuries to the spinal cord in its entirety or part [19]. These people may have large masses in their mediastinum, which makes it difficult for them to breathe. These tumours have the potential to induce scoliosis, respiratory problems, and cardiovascular consequences; however, our patient did not demonstrate any of these symptoms. A thoracic computed tomography (CT) scan and pulmonary function tests might be helpful, particularly in individuals who are already experiencing symptoms [7]. Before surgery, primary systemic hypertension and renal artery stenosis should also be evaluated. These conditions are among the most common indicators of cardiovascular impairment, thus they must be checked. Due to the presence of these symptoms, it is strongly recommended that nephrotoxic medicines be avoided before and during surgery. In the examination of hypertension, catecholamine levels in the urine and abdominal CT scans to look for pheochromocytomas, which are frequent in this group, should also be included [1]. Variations in blood pressure or cardiac arrhythmias should be flagged as potential indicators of a pheochromocytoma or carcinoid tumour during the perioperative phase. If the patient is not diagnosed before surgery due to the absence of clinical symptoms, he or she may develop an intraoperative hypertensive crisis as a result of surgical manipulation or the administration of trigger medications such as beta-blockers and ketamine. If this occurs, the patient will be hospitalised.

A mediastinal tumour can create vena cava compression and insufficient cardiac output during surgery, which can ultimately result in severe hypotension [20]. During the excision of a central nervous system (CNS) tumour, the anesthesiologist needs to keep the patient's blood pressure stable. Patients with NF1 have a much-increased risk of developing vasculopathy. Vasculopathies raise the risk of cerebral ischaemia as well as the possibility of aneurysm rupture [1]. In addition to this, the anesthesiologist has to be aware of the possibility of significant bleeding as a result of tumour invasion of surrounding tissues, as well as the presence of additional CNS malignancies that have not yet been discovered. When the patient has spinal abnormalities such as scoliosis or tumours of the spinal cord, doing a neuroaxial block, which is commonly used in pregnant women, might be problematic for the anesthesiologist [7]. Neuroaxial blocks are routinely used in pregnant women because they are safe and effective. Some research has been done to compare and contrast the characteristics of non-depolarizing and depolarizing neuromuscular blockers. The findings of this research suggest that neurofibromatosis patients may be able to get these medications in a risk-free manner. In conclusion, the case that was presented sheds insight into the anaesthetic features of an NF1 patient as well as the significance of proper anaesthetic care in ensuring a successful outcome.

## Conclusions

This case study highlights the need of recognising the specific anaesthetic needs of persons with neurofibromatosis type 1 who are having surgical procedures. The disorder known as neurofibromatosis is extremely uncommon and calls for the expertise of both a surgeon for its successful surgical correction and an anesthesiologist for successful perioperative management. Securing the patient's airway becomes difficult for the anesthesiologist because of the aberrant and excessive development of the tumour mass on the patient's face, specifically in our situation. To successfully induce and keep the patient under anaesthesia, a thorough preoperative evaluation and careful preparation are required. In conclusion, this case report illustrates the anaesthetic features of a patient with plexiform neurofibromatosis and highlights the need for adequate anaesthesia treatment for a successful outcome.
